# Supplementary material for: The Bordetella effector protein BteA induces host cell death by disruption of calcium homeostasis
Source: mBio. 2024 Nov 21;15(12):e01925-24. doi: 10.1128/mbio.01925-24 (PMC11633230; doi:10.1128/mbio.01925-24)
Supplement: Supplemental figures and tables — Fig. S1 to S10; Tables S1 to S3. [file mbio.01925-24-s0001.pdf]

SUPPLEMENTAL MATERIALS:

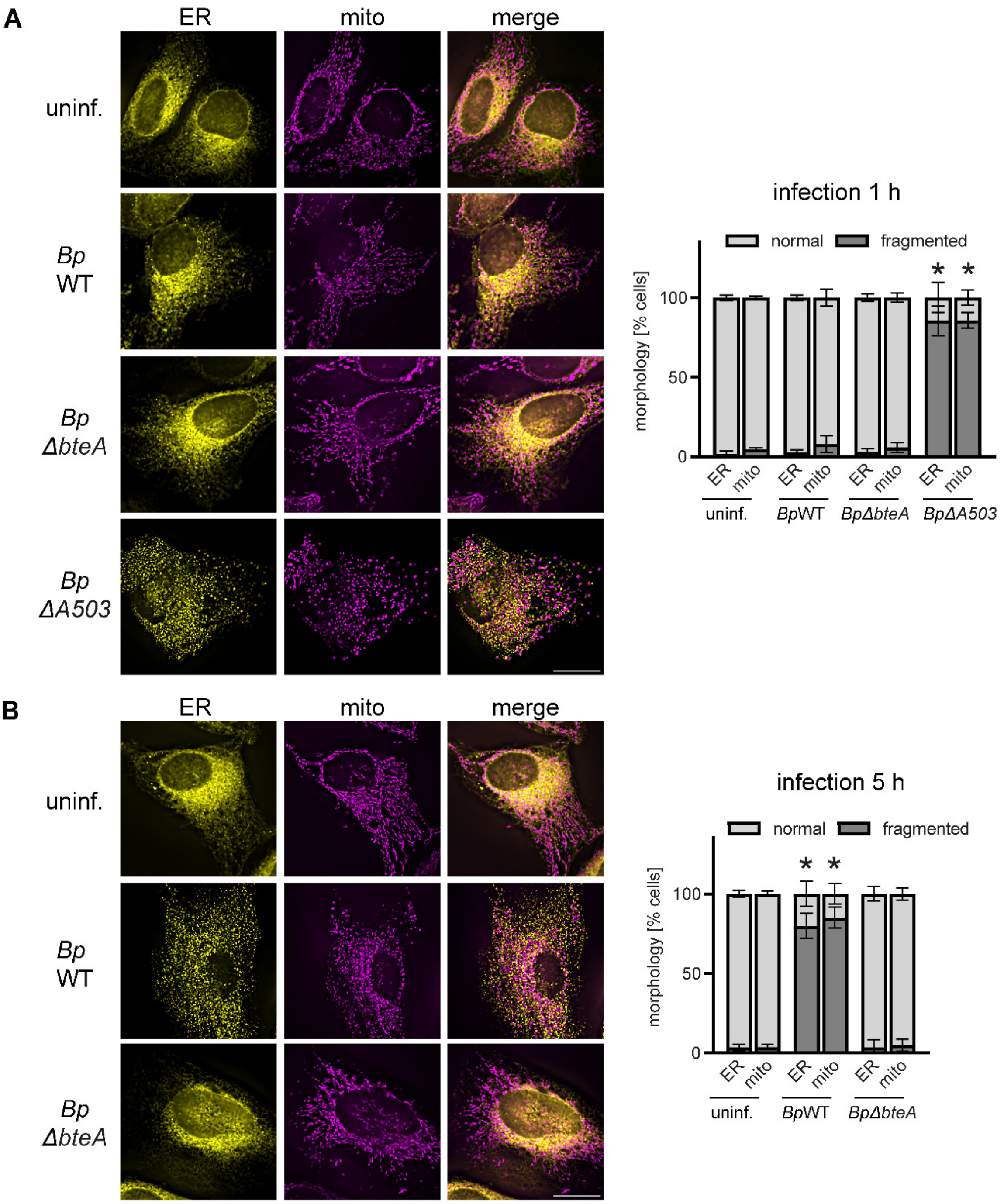

Figure S1. BteA-induced cell death is characterized by fragmentation of the endoplasmatic reticulum and mitochondrial networks.

Hela cells were transfected to express fluorescent proteins tagged with localization signals for endoplasmatic reticulum (ER) and mitochondria (mito). Cells were infected with the indicated *Bp* strains at an MOI 50:1 for 1 h **(A)** or 5 h **(B),** or left untreated, followed by fixation and analysis by fluorescence imaging. ER, yellow; mitrochondria, magenta. Scale bar, 20 µm. The shown micrographs are representative of 2 independent experiments from which the organelle morphology was scored. Analysis was performed on at least 100 cells per experiment and condition, and is plotted as morphology % ± SEM. Asterisks indicate statistically significant difference ($p < 0.05$, unpaired two-tailed *t*-test) between % of fragmented ER or mito compared with uninfected cells.

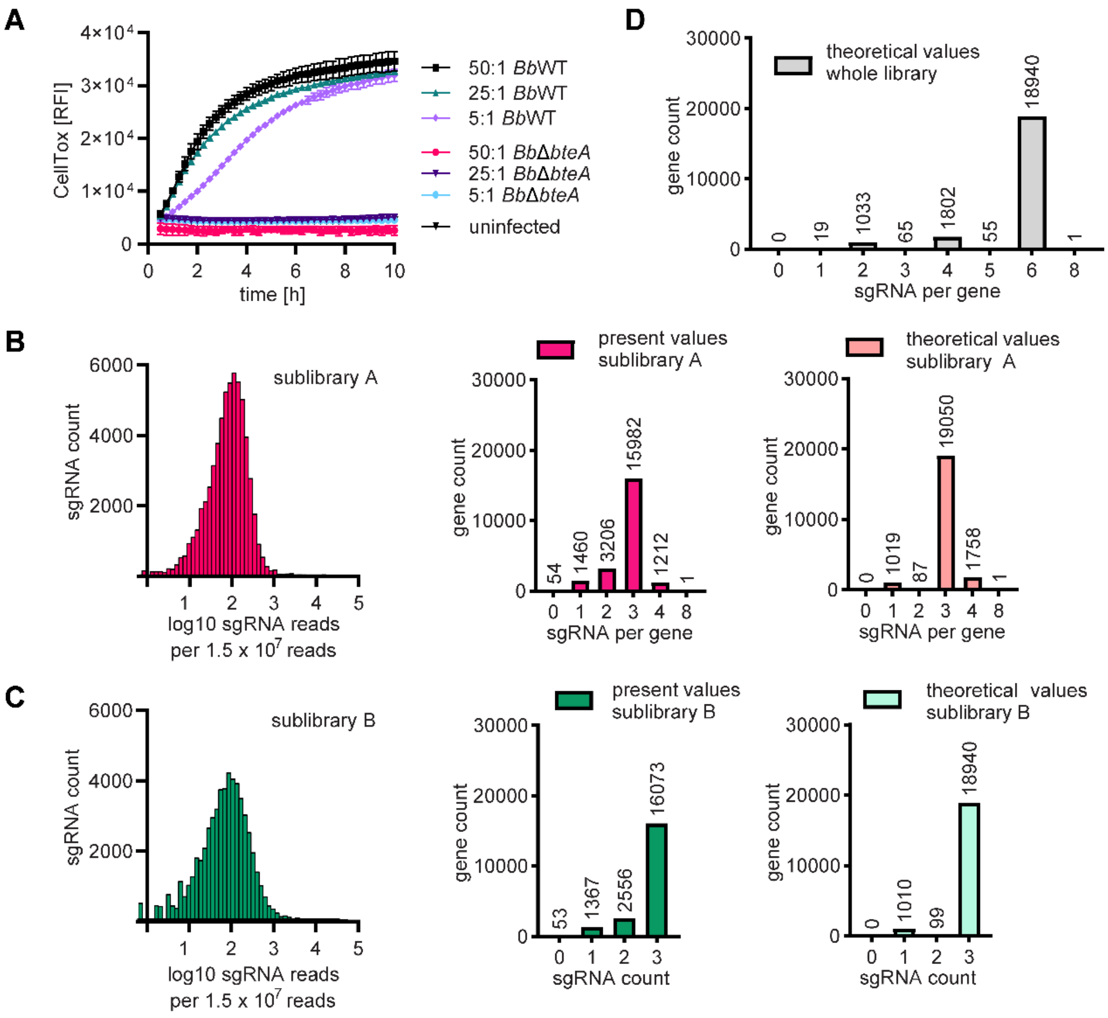

Figure S2. Susceptibility of HEK-Cas9 cells to *Bb*WT infection and characterization of HEK-Cas9 library complexity.

(A) Susceptibility of HEK-Cas9 cells**.** HEK-Cas9 cells were infected with *B. bronchiseptica* strains at the indicated MOI. Plasma membrane permeabilization was determined using the fluorescent DNA binding dye CellTox Green. Data represent the mean ± SEM of a representative experiment from 2 independent experiments performed in technical duplicate.

(B-C) Complexity in the individual HEK-Cas9 sublibraries A and B. Distribution of detected sgRNA is depicted in the histograms on the left. These histograms show the number of individual sgRNAs against their detection frequency. Non-detected sgRNA are shown as well. The middle graphs display the number of targeted genes per detected sgRNA count, and these are compared to the theoretically expected values shown in the right graphs.

(D) Theoretical complexity of the combined sublibraries A and B. The number of targeted genes per theoretical sgRNA count is indicated. Please compare with Fig. 2B in the main text.

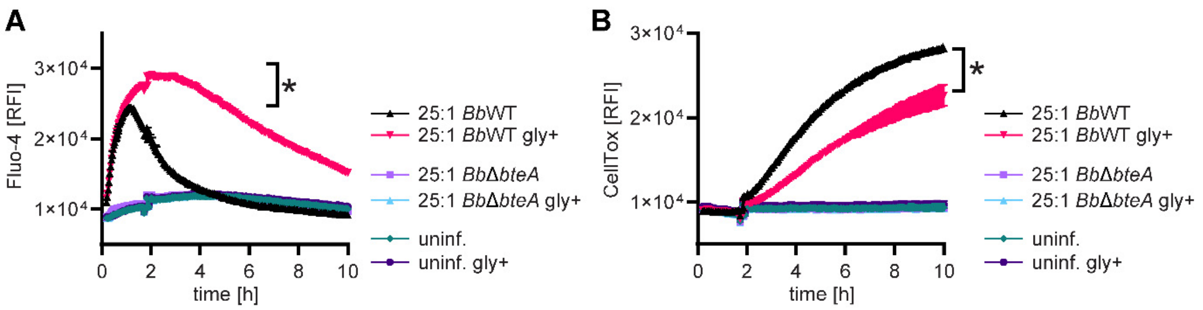

Figure S3. Inhibition of cell plasma membrane permeabilization by glycine does not diminish calcium influx.

HeLa cells were infected with *Bb*WT or *Bb*Δ*bteA* derivative at MOI 25:1 in the presence (gly+) or absence of 5 mM glycine. Calcium influx was assessed using Fluo-4/AM Ca^{2+} indicator **(A)** whereas plasma membrane permeabilization was determined in parallel wells by fluorescent DNA binding dye CellTox Green **(B)**. Asterisks indicate statistically significant difference ($p < 0.05$, unpaired two-tailed *t*-test) between *Bb*WT and *Bb*WT gly+ infection samples at 4 h. Difference between *Bb*WT and *Bb*WT gly+ at max *Bb*WT Fluo-4/AM (1 h) is not significant ($p > 0.05$). Data represent the mean ± SEM of a representative experiment from 2 independent experiments performed in technical duplicate.

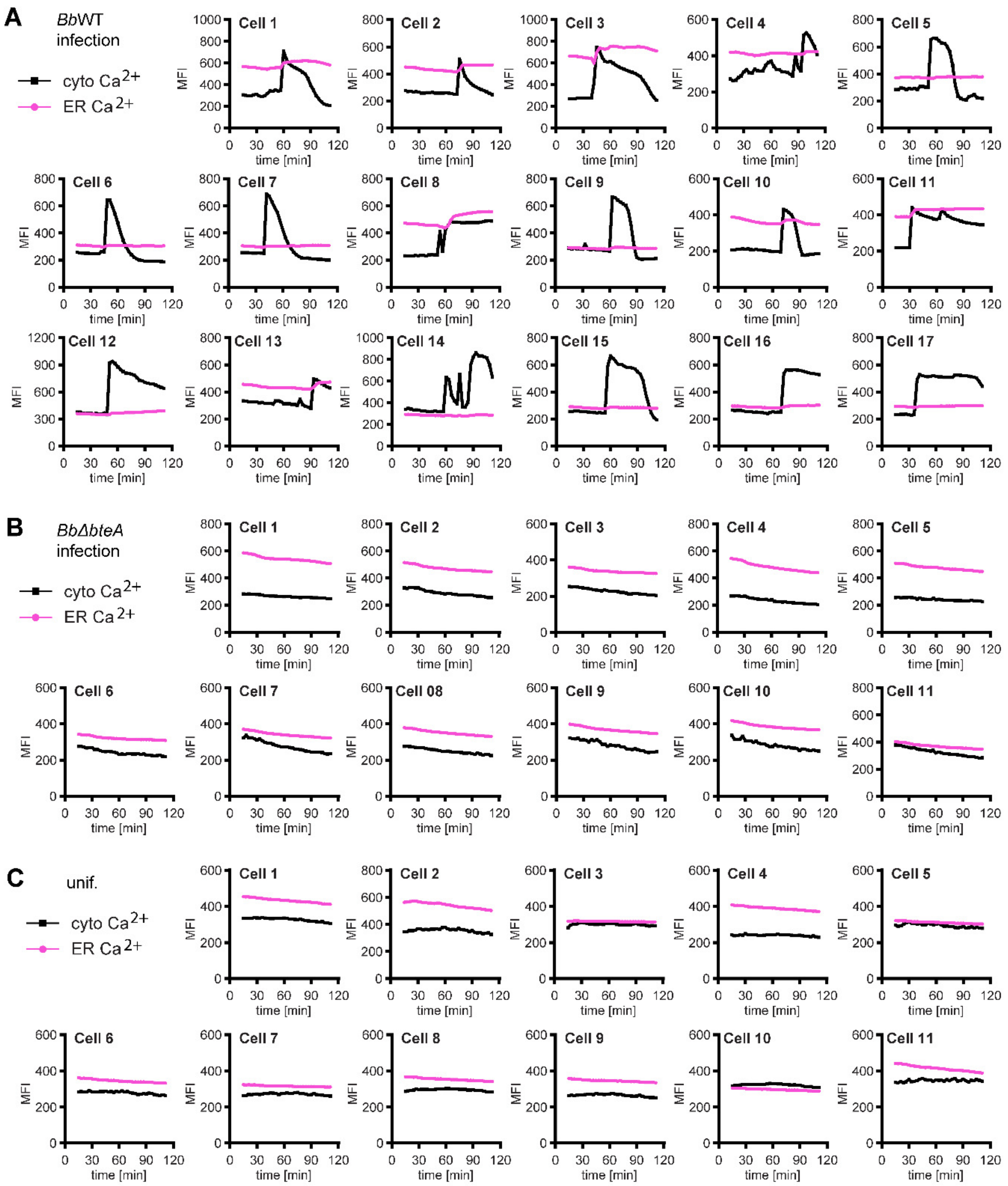

Figure S4. Correlation of cytosolic and ER calcium levels in individual cells.

Hela cells, transfected to express ER-targeted red Ca^{2+} sensor ER-LAR-GECO, were loaded with the cytosolic Ca^{2+} indicator Fluo-4/AM, and infected with *Bb*WT **(A)** or *Bb*Δ*bteA* **(B)** strains at MOI of 10:1, or left untreated **(C)**. The graphs indicate the mean fluorescence intensities of the individual cells quantified over time.

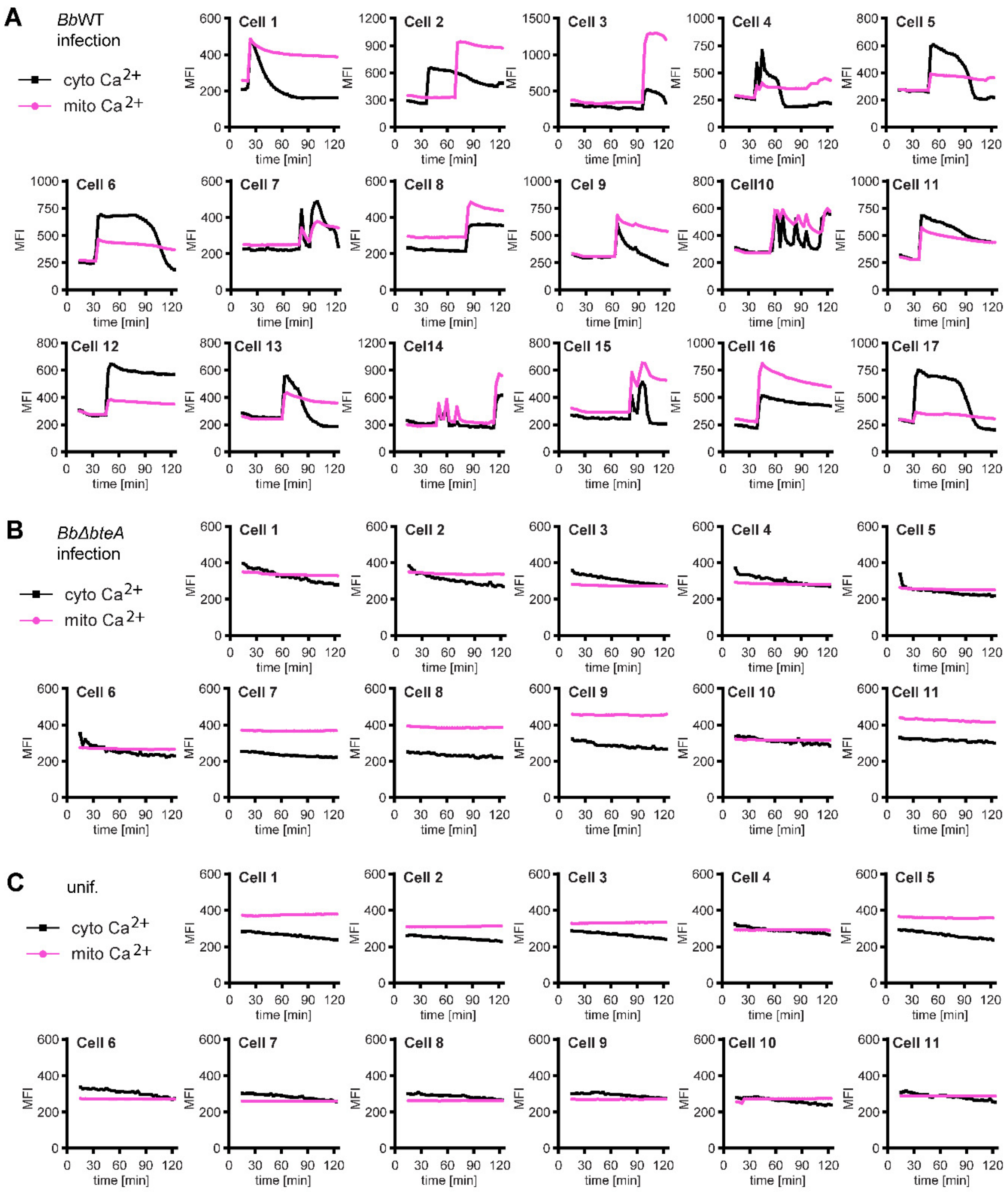

Figure S5. Correlation of cytosolic and mitochondrial calcium levels in individual cells.

Hela cells, transfected to express mitochondria-targeted red Ca^{2+} sensor mito-LAR-GECO, were loaded with the cytosolic Ca^{2+} indicator Fluo-4/AM, and infected with *Bb*WT **(A)** or *Bb*Δ*bteA* **(B)** strains at MOI of 10:1, or left untreated **(C)**. The graphs indicate the mean fluorescence intensities of the individual cells quantified over time.

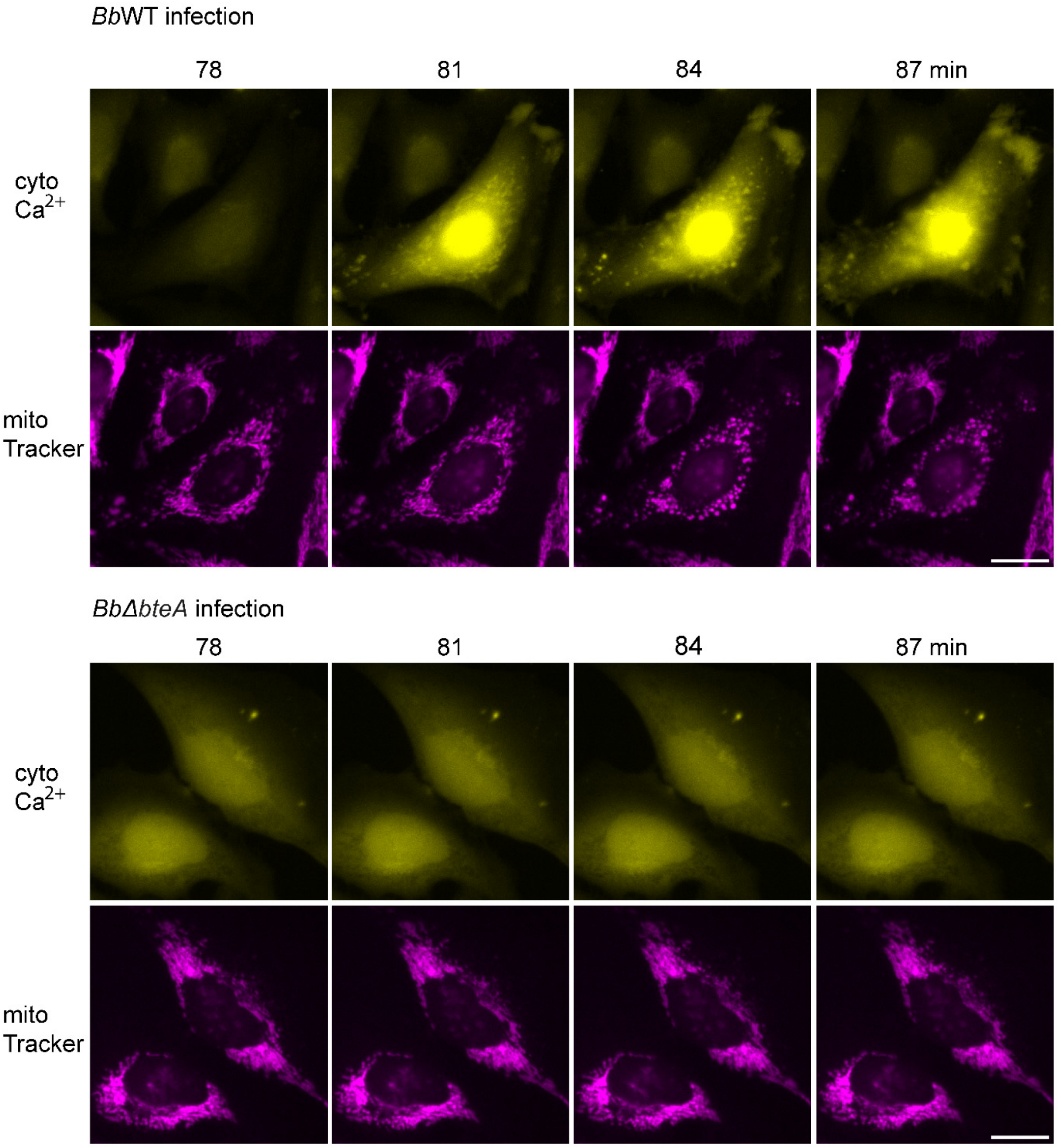

Figure S6. Assessment of mitochondrial morphology during *Bb* infection.

HeLa cells were loaded with MitoTracker, and the cytosolic Ca^{2+} indicator Fluo-4/AM, before being infected with *Bb*WT or *Bb*Δ*bteA* derivative at MOI 10:1. Sequence of time lapse images is shown. Data are representative of two independent experiments. Cytosolic Ca^{2+} indicator Fluo-4/AM, yellow; MitoTracker, magenta. Scale bar, 20 μm.

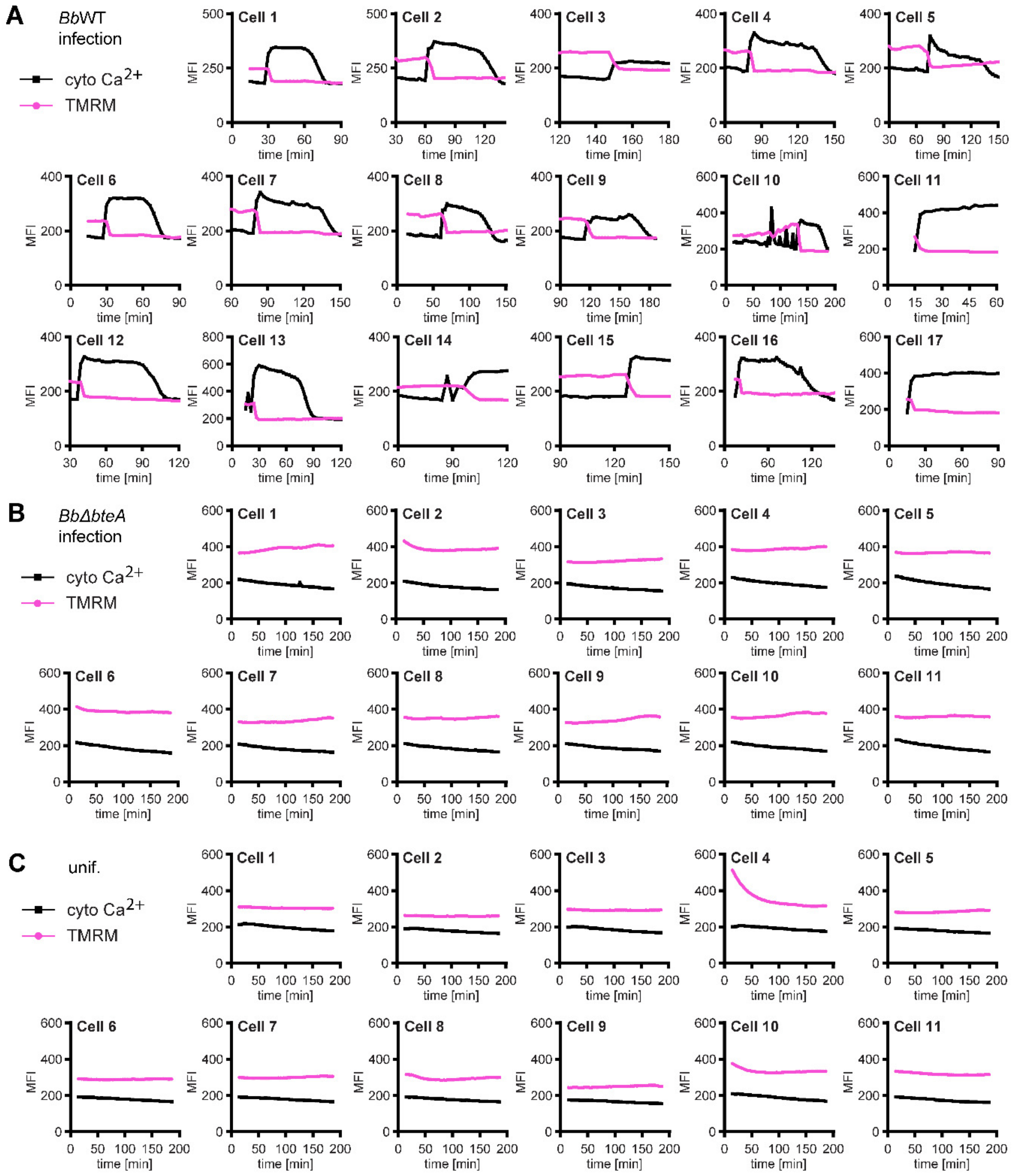

Figure S7. Correlation of cytosolic calcium levels and mitochondrial membrane potential in individual cells.

HeLa cells were loaded with the mitochondrial membrane potential indicator TMRM and the cytosolic Ca^{2+} indicator Fluo-4/AM**,** and infected with *Bb*WT **(A)** or *Bb*Δ*bteA* **(B)** strains at MOI of 10:1, or left untreated **(C)**. The graphs indicate the mean fluorescence intensities of the individual cells quantified over time.

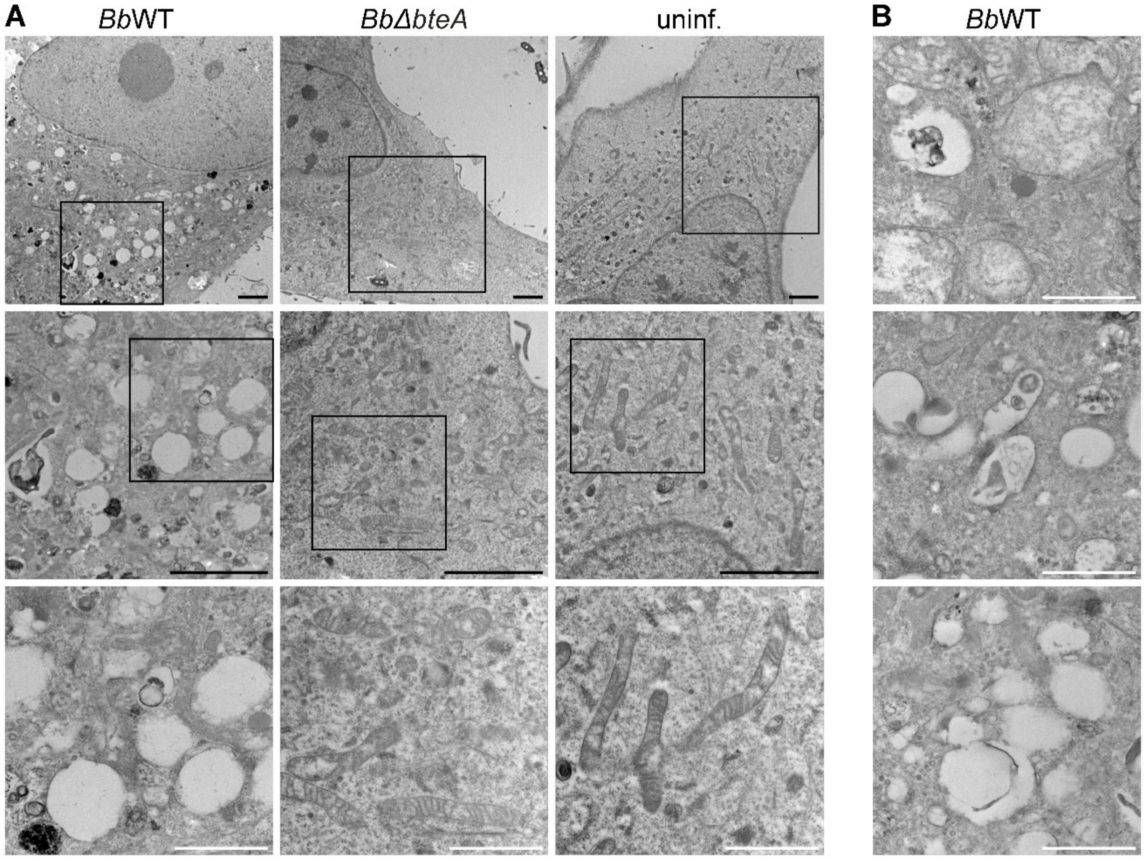

Figure S8. Transmission electron microscopy analysis.

Hela cells were infected with *Bb*WT and *BbΔbteA* at MOI of 25:1 for 1 h or left untreated. Following fixation, ultrathin sections were prepared and analyzed. In panel (A), black squares indicate the regions that are magnified. Additional images of *Bb*WT-infected cells are displayed in panel (B). Images are representative of two independent experiments. Black scale bar, 2 μm; white scale bar, 1 μm.

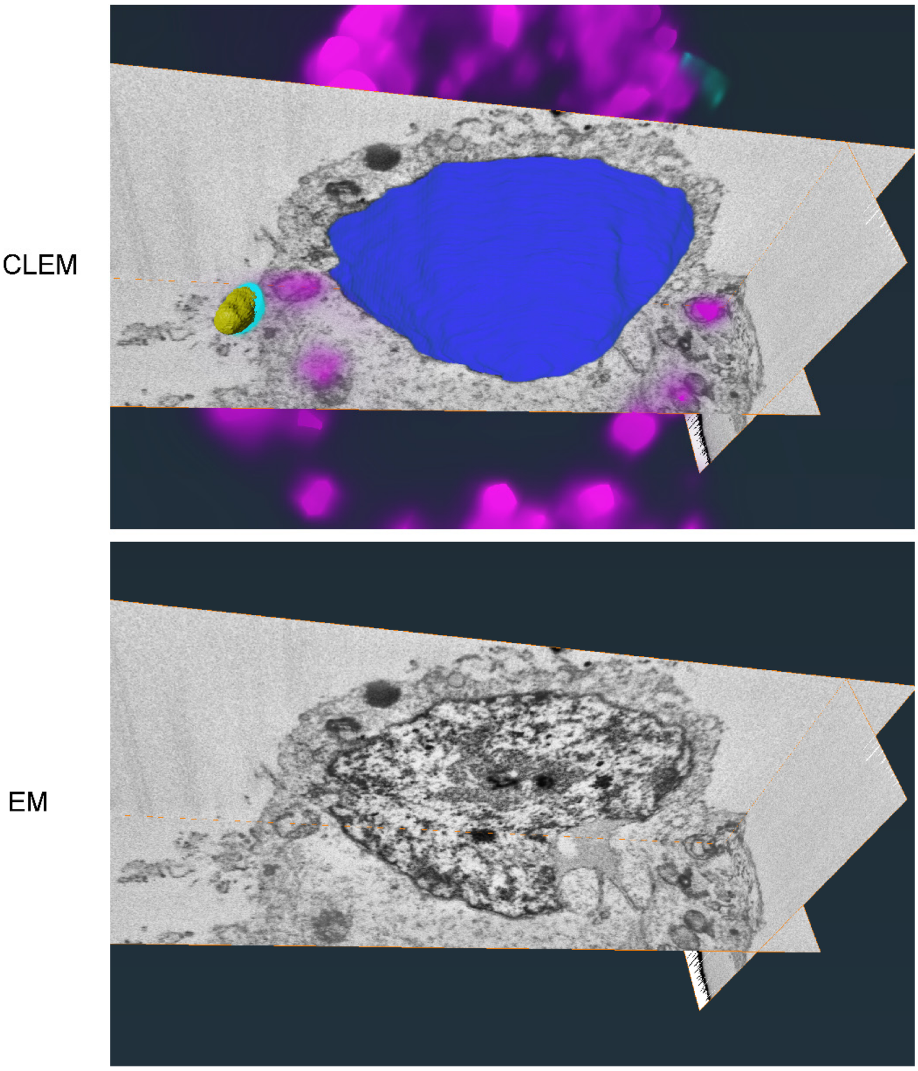

Figure S9. Correlative light-electron microscopy.

Hela cells, transfected to express mitochondria-targeted monomeric hyperfolder YFP fluorescent protein, were infected with mScarlet-expressing *B*bWT at MOI 10:1 for 0.5 h. Fluorescence imaging was performed during the fixation period to capture bacteria and mitochondrial structures. Subsequently, samples were prepared for electron microscopy and FIB-SEM imaging. Data was processed in Amira 3D 2024.1 software. Mitrochondria, magenta; bacteria, cyan/yellow; nuclei, blue.

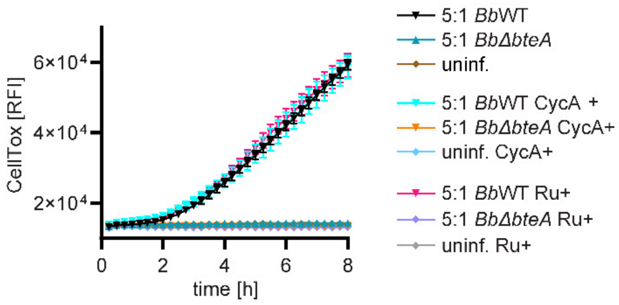

Figure S10. Role of mitochondria failure in the execution of BteA-induced cell death.

HeLa cells were pre-incubated with either 1 μM cyclosporin A (CycA+) or 10 μM ruthenium 360 (Ru+) for 1 h, or left untreated, before being infected with *Bb*WT and Δ*bteA* derivative at MOI 5:1. Plasma membrane permeabilization was determined using the fluorescent DNA binding dye CellTox Green. Data represent the mean ± SEM of a representative experiment from 2 independent experiments performed in technical triplicate.

Table S1. List of genes for which no sgRNA was detected in the combined sublibraries A and B despite their theoretical presence.

Gene_ID	Gene_name	Description
PLN	Phospholamban	Inhibitor of cardiac sarcoplasmic reticulum Ca^{2+} pump (1)
hsa-mir-1207	MicroRNA 1207	unknown
hsa-mir-1299	MicroRNA 1299	regulation of tumor pathogenesis, tumor suppressor (2)
hsa-mir-4679-1	MicroRNA 4679-1	unknown
hsa-mir-4679-2	MicroRNA 4679-2	unknown
hsa-mir-5096	MicroRNA 5096	unknown
hsa-mir-5591	MicroRNA 5591	unknown
hsa-mir-6088	MicroRNA 6088	unknown
hsa-mir-8078	MicroRNA 8078	unknown
NonTargetingControlGuide ForHuman_0583	-	Non-targeting control guide

Table S2. List of bacterial strains used in this study.

Strain	Genotype and relevant description	Internal number	Reference
***E. coli* strains**			
XL1-Blue	*rec*A1 *end*A1 *gyr*A96 *thi*-1 *hsd*R17 *sup*E44 *rel*A1 *lac* F´ *pro*AB *lacIq*ZΔM15 Tn10 Tet^r		Stratagene
SM10 λpir	*thi thr leu ton*A *lac*Y *sup*E *rec*A::RP4-2-Tc::Mu Km λpir		(3, 4)
***B. bronchiseptica* strains**			
*Bb*WT	*Bb*RB50 WT; wild type *Bordetella bronchiseptica* RB50 (B1976); complex I rabbit isolate; ST-12	BB012	(5, 6)
*Bb*Δ*bteA*	*BbRB50* Δ*bteA*; *Bb*RB50 strain derivative with *bte*A in-frame deletion of codons L2-A657	BB020	(7)
*Bb*WT / mNG	*BbRB50* WT harboring pBBRI-encoded mNeonGreen (mNG) fluorescent protein under the control of *Bb*RB50 GroES promoter (PgroES)	pBB119	This study
*Bb*Δ*bteA* / *mNG*	*BbRB50 ΔbteA* harboring pBBRI-encoded mNeonGreen (mNG) fluorescent protein under the control of *Bb*RB50 groES promoter (PgroES)	pBB121	This study
*Bb*WT / mSc	*BbRB50 WT* harboring pBBRI-encoded mSacrlet (mSc) under the control of *Bp*TohamaI BvgAS-regulated filamentous hemagglutinin (*fhaB* gene) promoter (PfhaB)	pBB039	This study
*Bb*Δ*bteA* / mSc	*BbRB50 ΔbteA* derivative harboring pBBRI-encoded mSacrlet (mSc) under the control of *Bp*TohamaI BvgAS-regulated filamentous hemagglutinin (*fhaB* gene) promoter (PfhaB)	pBB117	This study
***Bordetella pertussis* strains**			
*Bp*WT	*Bp*B1917 WT; wild type *Bordetella pertussis* 1917*; fim2-1, fim3-2, ptxP3, ptxA1, ptxB2, ptxC2, ptxD1, ptxE1, prn2*	BP001	(8, 9)
*Bp*Δ*bteA*	*Bp*B1917 Δ*bteA; Bp*B1917 strain derivative with *bteA* in-frame deletion of codons L2-A656	BP003	(10)
*Bp*ΔA503	*Bp*B1917 *bteA* ΔA503; *Bp*B1917 strain derivative with *bteA* in-frame deletion of codon A503	BP006	(10)

Table S3. List of plasmids used in this study.

Plasmid	Description	Reference
pBBRI MCS	*lacPOZ' mob*$^+$, broad-host cloning vector, Cm^R	(11, 12)
pBBRI-PgroES-mNG	pBBRI vector with *Bb*RB50 promotor GroES (PgroES) and coding sequence of the mNeonGreen protein (mNG)	This study
pBBRI-PfhaB-mSc	pBBRI vector with *Bp*TohamaI filamentous hemagglutinin promotor (pfhaB) and coding sequence of the mScarlet protein (mSc)	(13)
export&KDEL-mScarlet	ER-mScarletI, pEGFP-N1-derived vector encoding mScarlet fluorescent protein targeted to mitochondria, Addgene item # 137805	(14)
4xmts-mNeonGreen	4xmts-mNeonGreen, pEGFP-N1-derived vector encoding mNeonGreen fluorescent protein targeted to mitochondria, Addgene item # 98876	(14)
GeCKO v2 sublibrary A	Human CRISPR Knockout Pooled Library (GeCKO v2) in lentiGuide-Puro A, Catalog #1000000049, Addgene item #52959	(15)
GeCKO v2 sublibrary B	Human CRISPR Knockout Pooled Library (GeCKO v2) in lentiGuide-Puro B, Catalog #1000000049, Addgene item #52960	(15)
pCMV-VSV-G	Vector encoding envelope protein for producing lentiviral and MuLV retroviral particles, Addgene item #8454	(16)
psPAX2	2nd generation lentiviral packaging plasmid, Addgene item #12260	Addgene, Trono Lab unpublished
ER-LAR-GECO	CMV-ER-LAR-GECO1, low affinity red intensiometric genetically encoded Ca^{2+} indicator targeted to ER, Addgene item #61244	(17)
mito-LAR-GECO	CMV-mito-LAR-GECO1.2, low affinity red intensiometric genetically encoded Ca^{2+} indicator targeted to mitochondria, Addgene item #61245	(17)
mito-mhYFP	Mito-mhYFP, pcDNA3.1-derived vector encoding monomeric hyperfolder YFP fluorescent protein targeted to mitochondria, Addgene item #186526	(18)

References

1. Koss KL, Kranias EG. 1996. Phospholamban: a prominent regulator of myocardial contractility. Circ Res 79:1059-63.
2. Kaiyuan D, Lijuan H, Xueyuan S, Yunhui Z. 2021. The role and underlying mechanism of miR-1299 in cancer. Future Sci OA 7:FSO693.
3. Simon R, Priefer U, Pühler A. 1983. A Broad Host Range Mobilization System for In Vivo Genetic Engineering: Transposon Mutagenesis in Gram Negative Bacteria. Bio/Technology 1:784.
4. Skopova K, Tomalova B, Kanchev I, Rossmann P, Svedova M, Adkins I, Bibova I, Tomala J, Masin J, Guiso N, Osicka R, Sedlacek R, Kovar M, Sebo P. 2017. Cyclic AMP-Elevating Capacity of Adenylate Cyclase Toxin-Hemolysin Is Sufficient for Lung Infection but Not for Full Virulence of Bordetella pertussis. Infect Immun 85.
5. Cotter PA, Miller JF. 1994. BvgAS-mediated signal transduction: analysis of phase-locked regulatory mutants of Bordetella bronchiseptica in a rabbit model. Infect Immun 62:3381-90.
6. Diavatopoulos DA, Cummings CA, Schouls LM, Brinig MM, Relman DA, Mooi FR. 2005. Bordetella pertussis, the causative agent of whooping cough, evolved from a distinct, human-associated lineage of B. bronchiseptica. PLoS Pathog 1:e45.
7. Navarrete KM, Bumba L, Prudnikova T, Malcova I, Allsop TR, Sebo P, Kamanova J. 2023. BopN is a Gatekeeper of the Bordetella Type III Secretion System. Microbiol Spectr 11:e0411222.
8. Bart MJ, Zeddeman A, van der Heide HG, Heuvelman K, van Gent M, Mooi FR. 2014. Complete Genome Sequences of Bordetella pertussis Isolates B1917 and B1920, Representing Two Predominant Global Lineages. Genome Announc 2.
9. Bart MJ, Harris SR, Advani A, Arakawa Y, Bottero D, Bouchez V, Cassiday PK, Chiang CS, Dalby T, Fry NK, Gaillard ME, van Gent M, Guiso N, Hallander HO, Harvill ET, He Q, van der Heide HG, Heuvelman K, Hozbor DF, Kamachi K, Karataev GI, Lan R, Lutynska A, Maharjan RP, Mertsola J, Miyamura T, Octavia S, Preston A, Quail MA, Sintchenko V, Stefanelli P, Tondella ML, Tsang RS, Xu Y, Yao SM, Zhang S, Parkhill J, Mooi FR. 2014. Global population structure and evolution of Bordetella pertussis and their relationship with vaccination. MBio 5:e01074.
10. Bayram J, Malcova I, Sinkovec L, Holubova J, Streparola G, Jurnecka D, Kucera J, Sedlacek R, Sebo P, Kamanova J. 2020. Cytotoxicity of the effector protein BteA was attenuated in Bordetella pertussis by insertion of an alanine residue. PLoS Pathog 16:e1008512.
11. Kovach ME, Phillips RW, Elzer PH, Roop RM, 2nd, Peterson KM. 1994. pBBR1MCS: a broad-host-range cloning vector. Biotechniques 16:800-2.
12. Kovach ME, Elzer PH, Hill DS, Robertson GT, Farris MA, Roop RM, 2nd, Peterson KM. 1995. Four new derivatives of the broad-host-range cloning vector pBBR1MCS, carrying different antibiotic-resistance cassettes. Gene 166:175-6.
13. Klimova N, Holubova J, Streparola G, Tomala J, Brazdilova L, Stanek O, Bumba L, Sebo P. 2022. Pertussis toxin suppresses dendritic cell-mediated delivery of B. pertussis into lung-draining lymph nodes. PLoS Pathog 18:e1010577.
14. Chertkova AO, Mastop M, Postma M, van Bommel N, van der Niet S, Batenburg KL, Joosen L, Gadella TWJ, Okada Y, Goedhart J. 2020. Robust and Bright Genetically Encoded Fluorescent Markers for Highlighting Structures and Compartments in Mammalian Cells. bioRxiv doi:10.1101/160374:160374.
15. Sanjana NE, Shalem O, Zhang F. 2014. Improved vectors and genome-wide libraries for CRISPR screening. Nat Methods 11:783-784.
16. Stewart SA, Dykxhoorn DM, Palliser D, Mizuno H, Yu EY, An DS, Sabatini DM, Chen IS, Hahn WC, Sharp PA, Weinberg RA, Novina CD. 2003. Lentivirus-delivered stable gene silencing by RNAi in primary cells. RNA 9:493-501.
17. Wu J, Prole DL, Shen Y, Lin Z, Gnanasekaran A, Liu Y, Chen L, Zhou H, Chen SR, Usachev YM, Taylor CW, Campbell RE. 2014. Red fluorescent genetically encoded Ca2+ indicators for use in mitochondria and endoplasmic reticulum. Biochem J 464:13-22.
18. Campbell BC, Paez-Segala MG, Looger LL, Petsko GA, Liu CF. 2022. Chemically stable fluorescent proteins for advanced microscopy. Nat Methods 19:1612-1621.
