# Supplementary material for: The Bordetella effector protein BteA induces host cell death by disruption of calcium homeostasis
Source: mBio. 2024 Nov 21;15(12):e01925-24. doi: 10.1128/mbio.01925-24 (PMC11633230; doi:10.1128/mbio.01925-24)
Supplement: Video legends — Legends for Videos S1 to S8. [file mbio.01925-24-s0002.docx]

# Legends for Supplemental Vidoes:

**Video S1. Imaging of morphological changes and plasma membrane permeability in HeLa cells.** HeLa cells were infected with *B. bronchiseptica* wild type (*Bb*WT) and *Bb*Δ*bteA* mutant (*Bb*Δ*bteA*), expressing the fluorescent protein mNeonGreen, at MOI of 10:1 in the presence of propidium iodide (5 µg/ml). Bacteria were centrifugated onto cell surface. Images were captured every 5 min and are shown at 3 frames per second. Video is representative of three independent experiments. Bright field, gray; bacteria, cyan; propidium iodide, magenta. Scale bar, 20 µm.

**Video S2. Calcium imaging.** HeLa cells, loaded with cytosolic Ca^2+^ indicator Fluo-4/AM, were infected with *Bb*WT and *Bb*Δ*bteA,* which express the fluorescent protein mScarlet, at MOI of 10:1. To allow tracking bacterial attachment, no centrifugation of bacteria onto the cell surface was performed. Images were captured at 2 min intervals and are displayed at 3 frames per second. This video is representative of two independent experiments. Bacteria, magenta; cytosolic Ca^2+^ indicator Fluo-4/AM, yellow. Scale bar, 20 µm.

**Video S3. Calcium imaging.** HeLa cells, loaded with cytosolic Ca^2+^ indicator Fluo-4/AM, were infected with mScarlet-expressing *Bb*WT and *Bb*Δ*bteA* at MOI of 10:1. Bacteria were centrifugated onto cell surface. Images were captured every 2 min and are shown at 3 frames per second. Video is representative of three independent experiments. Bacteria, magenta; cytosolic Ca^2+^ indicator Fluo-4/AM, yellow. Scale bar, 20 µm.

**Video S4. ER calcium imaging.** HeLa cells, transfected to express red fluorescent Ca^2+^ sensor targeted to ER (ER-LAR-Geco), were loaded with cytosolic Ca^2+^ indicator Fluo-4/AM. Infection with *Bb*WT and *Bb*Δ*bteA* was performed at MOI 10:1, followed by bacterial centrifugation onto cell surface. Images were captured at 3 min intervals and are displayed at 3 frames per second. Video is representative of two independent experiments. Cytosolic Ca^2+^ indicator Fluo-4/AM, yellow; ER Ca^2+^ sensor, magenta. Scale bar, 20 µm.

**Video S5. Mitochondrial calcium imaging.** HeLa cells, transfected to express red fluorescent Ca^2+^ sensor targeted to mitochondria (mito-LAR-Geco), were loaded with cytosolic Ca^2+^ indicator Fluo-4/AM. Infection with *Bb*WT and *Bb*Δ*bteA* was performed at MOI 10:1, followed by bacterial centrifugation onto cell surface. Images were captured at 3 min intervals and are displayed at 3 frames per second. This video is representative of two independent experiments. Cytosolic Ca^2+^ indicator Fluo-4/AM, yellow; mitochondria Ca^2+^ sensor, magenta. Scale bar, 20 µm.

**Video S6. Imaging of mitochondria morphology.** HeLa cells were loaded with the MitoTracker and the cytosolic Ca*^2+^* indicator Fluo-4/AM, before being infected with *Bb*WT and *Bb*Δ*bteA* derivative at MOI 10:1. Bacteria were centrifugated onto cell surface. Images were captured at 3 min intervals and are displayed at 3 frames per second. Video is representative of three independent experiments. MitoTracker, magenta; cytosolic Ca^2+^ indicator Fluo-4/AM, yellow. Scale bar, 20 µm.

**Video S7. Imaging of mitochondrial membrane potential.** HeLa cells were loaded with the mitochondrial membrane potential probe TMRM, and the cytosolic Ca*^2+^* indicator Fluo-4/AM, before being infected with *Bb*WT and *Bb*Δ*bteA* derivative at MOI 10:1. Bacteria were centrifugated onto cell surface. Images were captured at 3 min intervals and are displayed at 3 frames per second. Video is representative of three independent experiments. TMRM, magenta; cytosolic Ca^2+^ indicator Fluo-4/AM, yellow. Scale bar, 20 µm.

**Video S8. Imaging of mitochondrial membrane potential.** HeLa cells were pre-incubated with the inhibitor of mitochondrial calcium uniporter ruthenium 360 (Ru360+) at 10 µM or left untreated. Subsequently, the cells were loaded with TMRM, and the cytosolic Ca*^2+^* indicator Fluo-4/AM, before being infected with *Bb*WT at MOI 10:1. Bacteria were centrifugated onto cell surface. Images were captured at 3 min intervals and are displayed at 3 frames per second. The video is representative of two independent experiments. TMRM, magenta; cytosolic Ca^2+^ indicator Fluo-4/AM, yellow. Scale bar, 20 µm.
